# Transcriptome and proteome quantification of a tumor model provides novel insights into post‐transcriptional gene regulation

**DOI:** 10.1186/gb-2013-14-11-r133

**Published:** 2013-11-30

**Authors:** Christoph Jüschke, Ilse Dohnal, Peter Pichler, Heike Harzer, Remco Swart, Gustav Ammerer, Karl Mechtler, Juergen A Knoblich

**Affiliations:** 1 Institute of Molecular Biotechnology of the Austrian Academy of Sciences (IMBA), Dr Bohr‐Gasse 3, 1030 Vienna, Austria; 2 Christian Doppler Laboratory for Proteome Analysis, Dr Bohr‐Gasse 9, 1030 Vienna, Austria; 3 Research Institute of Molecular Pathology (IMP), Dr Bohr‐Gasse 7, 1030 Vienna, Austria; 4 Thermo Fisher Scientific, Abberdaan 114, 1046 AA Amsterdam, Netherlands

## Abstract

**Background:**

Genome‐wide transcriptome analyses have given systems‐level insights into gene regulatory networks. Due to the limited depth of quantitative proteomics, however, our understanding of post‐transcriptional gene regulation and its effects on protein‐complex stoichiometry are lagging behind.

**Results:**

Here, we employ deep sequencing and the isobaric tag for relative and absolute quantification (iTRAQ) technology to determine transcript and protein expression changes of a *Drosophila* brain tumor model at near genome‐wide resolution. In total, we quantify more than 6,200 tissue‐specific proteins, corresponding to about 70% of all transcribed protein‐coding genes. Using our integrated data set, we demonstrate that post‐transcriptional gene regulation varies considerably with biological function and is surprisingly high for genes regulating transcription. We combine our quantitative data with protein‐protein interaction data and show that post‐transcriptional mechanisms significantly enhance co‐regulation of protein‐complex subunits beyond transcriptional co‐regulation. Interestingly, our results suggest that only about 11% of the annotated *Drosophila* protein complexes are co‐regulated in the brain. Finally, we refine the composition of some of these core protein complexes by analyzing the co‐regulation of potential subunits.

**Conclusions:**

Our comprehensive transcriptome and proteome data provide a valuable resource for quantitative biology and offer novel insights into understanding post‐transcriptional gene regulation in a tumor model.

## Background

Eukaryotic gene expression involves transcription, mRNA processing and decay, translation, and protein modification and degradation. Each of these steps is tightly regulated to ensure the proper function and stability of the biological system [1]. While genome and transcriptome data have accumulated rapidly since the advent of microarray and deep‐sequencing technologies, the limited depth of quantitative proteomics has inhibited similar progress in post‐transcriptional gene regulation. Therefore, transcript levels are still routinely used as the only measure for gene expression in high‐throughput approaches. Several studies, however, have reported a low correlation between transcript and protein levels [2]‐[6], highlighting the importance of post‐transcriptional processes as well as the limited predictive value of transcripts for protein expression. Hence, a better understanding of genetic information processing requires consideration of quantitative information at every step of gene expression control.

Recently, studies have begun to address this problem systematically by acquiring large‐scale quantitative mRNA and protein data from bacteria [7,8], yeasts [9]‐[11] and cell lines [12,13]. For complex tissues of higher organisms, however, such information is still rare. Quantitative analyses are either restricted to a few hundred genes due to limited proteome coverage [5,14] or they focus on cultured cell lines that might have lost properties of their tissue of origin over time [12,13,15]‐[17].

We therefore set out to address this problem using a complex neural tissue in wild‐type state and tumor state. The *Drosophila* brain arises from neural stem cells called neuroblasts that undergo repeated rounds of asymmetric cell division giving rise to self‐renewing neuroblasts and terminally differentiating neurons [18]‐[20]. In homozygous *brain tumor* (*brat*) mutants, some neuroblast divisions become symmetric leading to the formation of excess neuroblasts at the expense of neurons. This causes an uncontrolled expansion of the neuroblast pool and results in the formation of a large brain tumor [21]‐[23]. These tumors can be transplanted into host flies, where they become aneuploid and undergo metastasis [24]. Normally, tumor formation is lethal during larval development, but hypomorphic mutants can survive until adulthood, and the flies harbor large proliferating neuroblast tumors in their brains. The simple cytology of the developing *Drosophila* brain and the reproducibility of tumor formation have made *brat* mutants a well‐studied example for stem‐cell‐derived tumor formation.

Here, we performed an in‐depth integrative analysis of transcript and protein expression data from a complex metazoan tissue, comparing *Drosophila* brain tumor (*brat*) versus wild‐type heads. Using relative protein quantification with mass spectrometry (isobaric tag for relative and absolute quantification (iTRAQ)) [25], we determined relative expression levels for more than 6,200 proteins, corresponding to about 70% of all transcribed protein coding genes.

By investigating transcript–protein correlations, namely the change of correlation between the normal and tumorous state, we identify biological processes that are strongly regulated by post‐transcriptional mechanisms. Furthermore, we demonstrate that the stoichiometric expression of protein‐complex subunits is controlled by a two‐tiered mechanism involving co‐expression on the mRNA level followed by post‐transcriptional fine‐tuning. Surprisingly, our data suggest that co‐regulation of protein‐complex subunits is the exception and not the rule. Finally, our comprehensive data set provides a valuable resource for quantitative systems‐level analyses.

## Results and discussion

### About 60% of protein‐coding transcripts are expressed in wild‐type and *brat* fly heads

**Figure 1:**
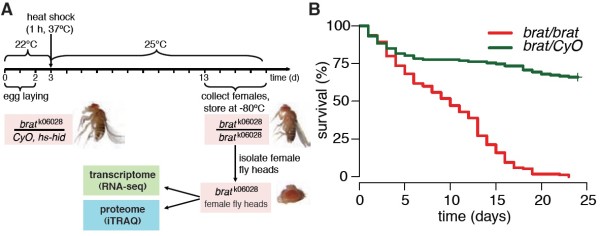
**Sample preparation workflow. (A)** Breeding scheme for generation of homozygous mutant fly heads. Fly eggs were collected over 2 days. On the third day, heterozygous offspring were killed by heat shock. Adult female flies were collected 1 to 3 days after pupal eclosion and snap‐frozen and their heads were isolated. **(B)** Kaplan–Meier survival plot of homozygous *brat* mutant (red) versus control flies (green). The tumor penetrance is 100% reducing the median adult survival time to 10 days. Data were pooled from three independent experiments with 170 *brat* and 163 control adult female flies in total.

To obtain sufficient amounts of material for transcriptome and proteome analyses we established a workflow to collect large numbers of homozygous *brat* mutant fly heads (Figure 1A). Homozygous mutant female flies exhibited a tumor penetrance of 100%, and the median adult survival time was reduced to 10 days (Figure 1B).

**Figure 2:**
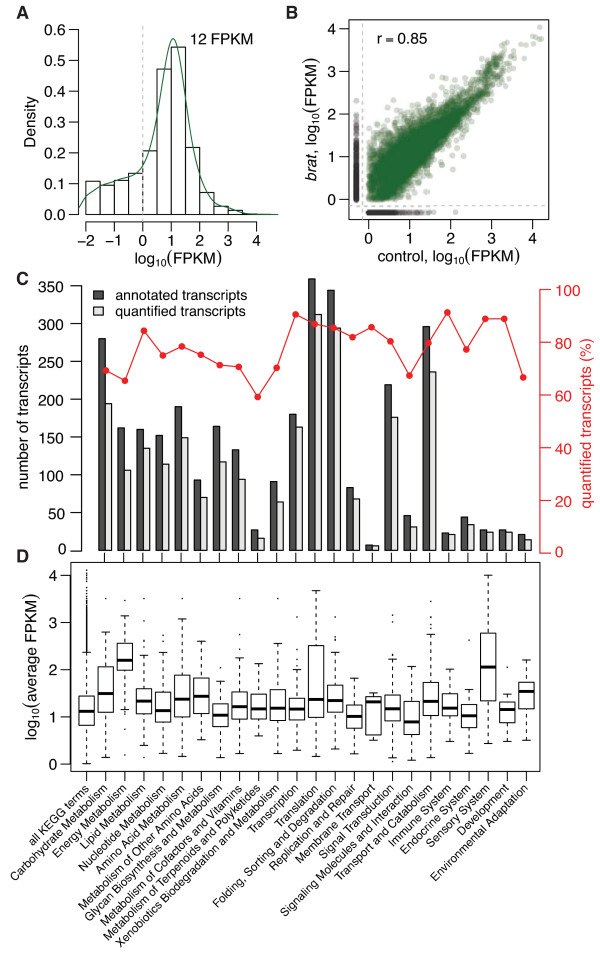
**Transcriptome analysis. (A)** Distribution of the raw FPKM data (green). Transcripts with FPKM ≥ 1 (dashed gray line) are considered expressed and functional. The majority of transcripts have FPKM values of about 12. **(B)** Absolute transcript levels in *brat* and wild‐type samples correlate well. **(C)** About 82% of all KEGG‐annotated transcripts are expressed in fly heads. For each functional category the number of annotated (dark gray) and quantified transcripts (light gray) are shown together with the percentage of quantified transcripts (red). **(D)** Box‐and‐whisker plot of the average transcript abundance (FPKM) in different KEGG categories. FPKM, fragments per kilobase of transcript per million mapped fragments; KEGG, Kyoto Encyclopedia of Genes and Genomes.

For transcriptome analysis, total RNA samples from *brat* and wild‐type female fly heads were prepared in biological triplicates, analyzed by strand‐specific paired‐end mRNA sequencing and quantified by mapping the reads to the *Drosophila* genome. The average expression levels (measured as fragments per kilobase of transcript per million mapped fragments (FPKM)) showed a bimodal distribution with most genes following a normal distribution centered at approximately 12 FPKM, and a minority forming a ‘shoulder’ to the left of the distribution (Figure 2A). Transcripts in the left shoulder with FPKM < 1 were shown to occur in less than one copy per cell and to have functions not typical for the cell type [26]. Hence, we excluded these very low abundant, presumably non‐functional, transcripts from our analysis.

We found that transcript expression correlated well between wild‐type and *brat* samples (Pearson correlation coefficient *r*=0.85, Figure 2B) indicating that the tumors maintain many characteristics of the corresponding wild‐type tissue. In total, we were able to quantify transcripts from 8,333 of the 13,781 annotated protein‐coding *Drosophila* genes in both wild‐type and *brat* mutant heads. On average 82% of all annotated transcripts were expressed in adult female fly heads for each second‐level Kyoto Encyclopedia of Genes and Genomes (KEGG) category [27] (Figure 2C). The highest absolute mRNA expression levels were found in the categories ‘Energy Metabolism’ and ‘Translation’, which agrees well with recent data from fission yeast [11], and in the category ‘Sensory System’, consistent with the specific functions of the analyzed tissue (Figure 2D).

### Quantification of approximately 70% of the brain tumor proteome

In a pilot proteomic study, *brat* and wild‐type fly head samples were labeled in duplicate with 4‐plex iTRAQ [25], separated by two‐dimensional liquid chromatography and measured by online tandem mass spectrometry. We quantified expression changes of 68,391 peptides with 8,017 unique sequences, corresponding to 1,311 unique proteins at a protein false discovery rate (FDR) of 5%. We refer to this first data set as iTRAQ #1.

Taking the number of quantified protein‐coding transcripts as an estimate for the total number of expressed proteins, we set out to increase the proteome coverage of our iTRAQ analysis. For this, we optimized the proteomics workflow by employing digestion with two proteases, high‐resolution two‐dimensional chromatography with extensive fractionation, combined collisional‐induced dissociation (CID)/higher energy C‐trap dissociation (HCD) and electron transfer dissociation (ETD)/HCD fragmentation [28], and multiple search engines using Protein Discoverer (Thermo Fisher Scientific). In all further analyses and discussion we refer to this as the optimized iTRAQ data set (iTRAQ #2).

**Figure 3:**
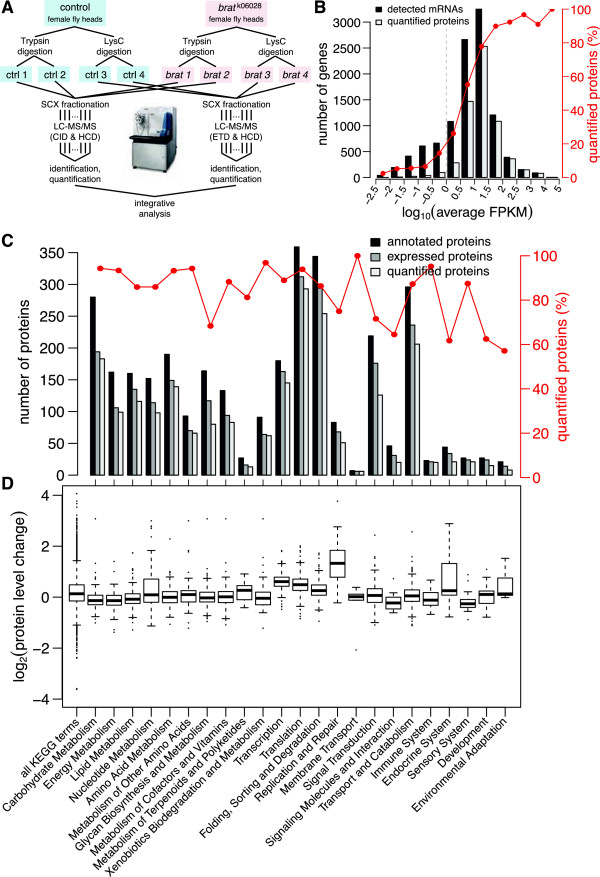
**Proteome analysis. (A)** Optimized mass spectrometry workflow for quantitative proteomics using 4‐plex iTRAQ. **(B)** Proteome coverage of expressed transcripts. The red line indicates the percentage of quantified proteins in each bin of detected transcripts. Transcripts with FPKM ≥ 1 are considered expressed (dashed gray line, see also Figure 2A). **(C)** Proteome coverage of different KEGG categories. For each functional category the number of annotated (dark gray), expressed (light gray) and quantified proteins (white) are shown together with the percentage of quantified proteins (red). **(D)** Box‐and‐whisker plot of protein expression changes between wild‐type and *brat* samples in different functional categories. FPKM, fragments per kilobase of transcript per million mapped fragments; KEGG, Kyoto Encyclopedia of Genes and Genomes.

Each sample was digested separately with two specific proteolytic enzymes, trypsin and LysC, and labeled in duplicate with 4‐plex iTRAQ. Trypsin‐and LysC‐digested samples were fractionated by high‐resolution strong cation exchange (SCX) chromatography with a two‐dimensional gradient into 85 and 118 fractions, respectively, and then analyzed by liquid chromatography‐tandem mass spectrometry (LC‐MS/MS) on a LTQ‐Orbitrap Velos (Figure 3A). Protein quantification of technical iTRAQ replicates correlated very well (*r*=0.99, Additional file 1: Figure S1A).

To confirm the iTRAQ data, we quantified 34 proteins by selected reaction monitoring (SRM) [29], an alternative label‐free protein quantification method. We observed a high technical and biological reproducibility of iTRAQ and SRM protein quantification (*r*=0.83, Additional file 1: Figure S1B and *r*=0.73, Additional file 1: Figure S1C, respectively). In addition, a comparison of iTRAQ and SRM measurements showed that the level of regulation appeared higher for SRM than for iTRAQ. This is in agreement with previous observations reporting an underestimation of protein expression changes (‘ratio compression’) for iTRAQ [30,31]. To account for this effect, we performed correlation analyses between the different data sets using rank correlations.

In total, we were able to determine the relative expression of 278,763 peptides (FDR < 1.6%) containing 65,742 unique sequences, with 166,615 (60%) of the peptides from trypsin‐digested and 112,148 (40%) from LysC‐digested samples. The trypsin and LysC samples were largely complementary in their contribution of unique peptides for quantification (Additional file 1: Figure S1D).

The peptide sequences mapped unambiguously to 6,277 FlyBase‐annotated protein‐coding genes at a protein FDR of 5%. The FDR of the integrated data set was lower since we combined the proteome with transcriptome data and performed correlation analyses only for genes with both quantitative protein and transcript data available. Expression changes for 75% of the proteins were determined in both trypsin‐ and LysC‐digested samples (Additional file 1: Figure S1E), and showed good reproducibility (*r*=0.7, Additional file 1: Figure S1F). We found that 18% of the proteins were exclusively quantified in the trypsin sample and 7% in the LysC sample.

For 93% (5,840 of 6,277) of all quantified proteins we were able to quantify the corresponding transcripts as well, and the correlation between mRNA and protein expression changes was very similar to a previous study in cell lines (Spearman’s rank correlation *ρ*=0.61 versus *ρ*=0.58 to 0.63 in [12]). Considering that 8,333 protein‐coding genes were expressed in the samples according to transcriptome analysis, we have quantified 70% (5,840 of 8,333) of all expressed proteins. Therefore, our data represent one of the most complete quantitative proteomics analyses of a complex tissue comparing two physiological states.

### High quantitative proteome coverage for Kyoto Encyclopedia of Genes and Genomes pathways and abundant transcripts

To further evaluate the quality of our data, we correlated proteome coverage with expression levels, physico‐chemical properties and annotated functions. Using the codon adaptation index as a predictor for expression levels [32], we found increased coverage for proteins predicted to be more abundant (Additional file 2: Figure S2A). Furthermore, we observed increased coverage for proteins encoded by more abundant transcripts. For mRNAs with FPKM > 10, for example, we obtained a protein coverage of more than 82% (Figure 3B). As shown previously [33], we detected a higher proteome coverage for larger proteins since they generally produce a larger number of different peptides upon digestion (Additional file 2: Figure S2B). Only extremely hydrophobic proteins were under‐represented and covered by less than 50%, presumably due to reduced solubility during sample preparation (Additional file 2: Figure S2C), whereas protein coverage was higher than 60% for the full range of isoelectric points (Additional file 2: Figure S2D). Most importantly, our analysis quantified on average 86% of the expressed proteins within each second‐level KEGG pathway category with only five categories covered by less than 70% (Figure 3C). Thus, all biological pathways are well represented and our data are a good representation of the entire expressed proteome.

### DNA replication and damage repair pathways are upregulated in tumors

To identify deregulated biological processes in the tumors, we performed functional pathway enrichment analyses for transcript and protein level changes using a *z*‐value cut‐off of 2. The set of pathways over‐represented in upregulated transcripts and proteins largely overlapped (KEGG‐term enrichment analysis of upregulated transcripts and proteins: Tables 1 and 2, respectively). As expected for proliferating tumor tissue, the KEGG pathway ‘DNA replication’ was strongly enriched among proteins upregulated in *brat* (Table 2). Surprisingly, however, this list also contained multiple terms associated with DNA damage repair like ‘Mismatch repair’, ‘Nucleotide excision repair’ and ‘Base excision repair’ (Table 2), and proteins in the KEGG category ‘Replication and repair’ were most highly upregulated in the tumors (Figure 3D). Although genomic instability does not cause brain tumor formation in *Drosophila*[34], our results suggest that the DNA damage reported for *brat* tumors upon transplantation [24] might already be present in the primary tumor.

**Table 1:** Kyoto Encyclopedia of Genes and Genomes based enrichment analysis of upregulated transcripts

**Kyoto Encyclopedia of Genes**	**Enrichment**	***P *** **value**	**Adjusted**
**and Genomes pathway**			***P *** **value**
Replication and repair	18.4	1.3×10^−32^	2.05×10^−30^
Genetic information processing	4.19	1.92×10^−29^	1.52×10^−27^
DNA replication	21	6.03×10^−17^	3.18×10^−15^
Nucleotide excision repair	15.5	6.69×10^−12^	2.64×10^−10^
Mismatch repair	22.6	5.33×10^−11^	1.68×10^−9^
RNA transport	6.87	1.16×10^−10^	3.05×10^−9^
Transcription	5.3	1.21×10^−9^	2.72×10^−8^
Translation	3.59	9.41×10^−9^	1.86×10^−7^
Pyrimidine metabolism	6.56	8.27×10^−7^	1.45×10^−5^
Basal transcription factors	10	9.61×10^−7^	1.52×10^−5^
Non‐homologous end‐joining	31.8	2.75×10^−6^	3.95×10^−5^
Homologous recombination	13.2	2.68×10^−5^	0.000353
Base excision repair	11.9	4.69×10^−5^	0.00057
Ribosome biogenesis in eukaryotes	5.05	7.07×10^−5^	0.000798
Spliceosome	4.18	0.00014	0.00148
Progesteron‐mediated oocyte maturation	6.5	0.000292	0.00271
Endocrine system	6.5	0.000292	0.00271
Nucleotide metabolism	3.45	0.000361	0.00317
Organismal systems	3.33	0.00158	0.0131

**Table 2:** Kyoto Encyclopedia of Genes and Genomes based enrichment analysis of upregulated proteins

**Kyoto Encyclopedia of Genes**	**Enrichment**	***P *** **value**	**Adjusted**
**and Genomes pathway**			***P *** **value**
Replication and repair	19.5	6.32×10^−26^	9.99×10^−24^
DNA replication	34.3	2.17×10^−24^	1.72×10^−22^
Nucleotide excision repair	19.2	5.58×10^−12^	2.94×10^−10^
Homologous recombination	28.8	1.09×10^−10^	4.32×10^−9^
Mismatch repair	27.2	1.87×10^−10^	5.89×10^−9^
Base excision repair	22.6	1.25×10^−8^	3.29×10^−7^
Genetic information processing	2.56	1.63×10^−7^	3.69×10^−6^
Pyrimidine metabolism	8.09	4.04×10^−7^	7.98×10^−6^
Non‐homologous end‐joining	32.3	7.04×10^−5^	0.00124
Nucleotide metabolism	4.26	0.000125	0.00198
Progesterone‐mediated oocyte maturation	7.35	0.00056	0.00737
Endocrine system	7.35	0.00056	0.00737
Purine metabolism	3.54	0.00373	0.0453

### Hydrophobic proteins tend to be downregulated in the tumors

**Figure 4:**
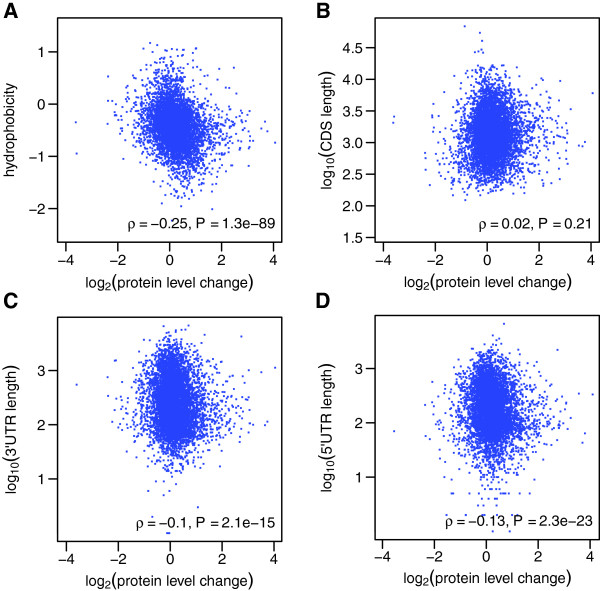
**Correlation of sequence features with changes in protein expression. (A)** Protein hydrophobicity negatively correlates with protein level change. **(B)** Length of the coding sequence does not affect protein expression changes. **(C)** Lengths of the 3’ and **(D)** 5’ UTRs negatively correlate with protein upregulation. CDS, coding sequence; UTR, untranslated region.

Our comprehensive data set of protein expression changes between wild‐type and *brat* samples allowed us to test for correlations with specific primary sequence features (Figure 4). Protein expression changes neither correlated with molecular weight (*ρ*=0.02) nor isoelectric point (*ρ*=0, data not shown). However, hydrophobic proteins were preferentially downregulated in the tumor (*ρ*=−0.25, *P*=1.3×10^−89^, Figure 4A). A possible explanation is that *brat* mutant neuroblasts fail to differentiate into neurons and hence do not upregulate the multitude of transmembrane proteins required for mature neuronal function [23,35,36].

### Shorter 3’ and 5’ UTRs correlate with protein upregulation

While protein level changes did not correlate with the length of coding sequences (*ρ*=0.02, Figure 4B), we detected a very low but significant negative correlation with the length of the 3’ and 5’ untranslated regions (3’ UTR: *ρ*=−0.1, *P*=2.1×10^−15^, Figure 4C; 5’ UTR: *ρ*=−0.13, *P*=2.3×10^−23^, Figure 4D). To determine if up‐ and downregulated proteins were differentially affected by UTR length, we divided the pool of quantified proteins into two subsets: downregulated and upregulated relative to the median, and analyzed the correlation for these subsets separately. Interestingly, only upregulated proteins with shorter UTR length were more highly upregulated (3’ UTR: *ρ*=−0.12, *P*=1.8×10^−10^; 5’ UTR: *ρ*=−0.11, *P*=3.5×10^−9^), whereas downregulated proteins did not have a significant correlation with UTR length. On average, transcripts with shorter UTRs are expected to have less binding sites for regulatory factors like miRNAs and RNA‐binding proteins, and thus, are less susceptible to post‐transcriptional control. Our observation is consistent with data showing that 3’ UTR shortening increases mRNA stability and protein expression, and leads to oncogene activation in cancer cells [37,38]. Interestingly, our data suggest that this effect might occur not only for 3’ UTRs but also for 5’ UTRs.

### Genes involved in transcription are strongly regulated by post‐transcriptional control

Next, we considered whether mRNA abundance had an effect on the expression change of proteins in the tumor. Interestingly, we observed a negative correlation of wild‐type transcript abundance with protein level change (*ρ*=−0.29, *P*=2.5×10^−118^, Additional file 3: Figure S3A), whereas the opposite was not the case: transcript levels in the tumor did not correlate with protein level change (Additional file 3: Figure S3B). Our results indicate that proteins encoded by transcripts, which are lowly expressed in wild‐type samples, have an increased propensity for being upregulated in the tumors.

**Figure 5:**
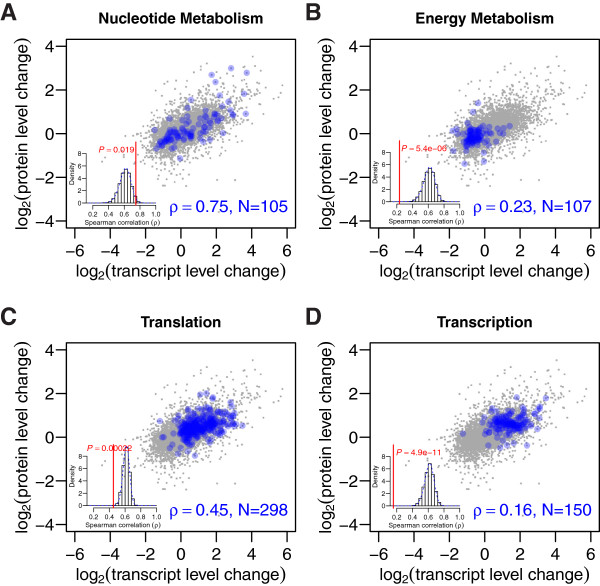
**Post‐transcriptional regulation changes for distinct functional pathways.** Transcript and protein level changes exhibit different correlations for distinct gene functions. Correlation *ρ* of transcript and protein level changes for genes involved in **(A)** nucleotide metabolism, **(B)** energy metabolism, **(C)** translation and **(D)** transcription. *N* is the number of quantified gene products in each category. The insets on the lower left side of each panel show histograms of correlation coefficients that were generated by random sampling (sample size *N*). The observed correlation coefficients and the corresponding *P* values are indicated in red.

Quantification of both mRNA and protein level changes allowed us to test the contribution of post‐transcriptional mechanisms to the proteome alterations that occur in *brat* brain tumors. For this, we compared how well changes in protein levels correlate with changes in the corresponding transcripts. Overall protein and mRNA changes correlated similarly to a previous analysis in human cell lines (*ρ*=0.61 versus *ρ*=0.58 to 0.63 in [12]). Surprisingly, when investigating the different biological pathways individually, we found that the correlations were highly variable. We used random sampling to control for the different number of genes in each KEGG category to identify categories that deviate significantly from the global correlation (Table 3 and Figure 5).

**Table 3:** Biological pathways exhibiting significant alterations in post‐transcriptional regulation

**Kyoto Encyclopedia of**	**Correlation *ρ***	**Significance**^**a**^
**Genes and Genomes pathway**		
**Metabolism**		
Energy metabolism	0.23	***
Lipid metabolism	0.49	*
Nucleotide metabolism	0.75	*
**Genetic information processing**		
Transcription	0.16	***
Translation	0.45	***
Folding, sorting and degradation	0.46	***
**Cellular processes**		
Transport and catabolism	0.39	***

^a^Different biological pathways show considerable variability in their correlation of transcript‐to‐protein level changes. The statistical significance of the deviation from the global correlation (*ρ*=0.61) was estimated by random sampling, controlling for the number of quantified proteins in each pathway (* *P*<0.05; ** *P*<0.01; *** *P*<0.001).

For KEGG pathways involved in ‘Metabolism’, the correlations did not show a clear trend. The correlation was very high for ‘Nucleotide metabolism’ (*ρ*=0.75,*N*=105, Figure 5A), indicating that changes of transcript expression cause corresponding changes in protein expression and, hence, only minor alterations in the post‐transcriptional regulation occur between wild‐type and tumor samples. In contrast, the correlation was low for ‘Energy metabolism’ (*ρ*=0.23,*N*=107, Figure 5B). Here, however, transcript and protein expression levels were relatively constant and therefore no conclusions about changes in post‐transcriptional regulation were possible.

The KEGG subcategories for ‘Genetic information processing’ generally showed significantly lower correlations. The correlation was low for ‘Translation’ (*ρ*=0.45,*N*=298, Figure 5C) and lowest for genes regulating ‘Transcription’ (*ρ*=0.16,*N*=150, Figure 5D). To control for the particular spread of mRNA and protein regulation, respectively, we compared the correlation of ‘Transcription’ genes to randomly sampled genes exhibiting similar spreads and found that the observed correlation was significantly lower than what would be expected by chance (data not shown). Thus, proteins involved in transcriptional processes are particularly well controlled on a post‐transcriptional level, and changes of mRNA expression provide only limited insight into changes of protein expression. This is important to consider when performing quantitative research on the regulation of transcription: our data indicate that quantifying only mRNA expression might not always suffice to reflect the situation at the protein level.

### Co‐regulation of protein complexes is enhanced post‐transcriptionally

Most proteins exert their biological functions as part of supramolecular assemblies and complexes, and much progress has been made in identifying these protein complexes on a global scale [39]‐[41]. While transcripts coding for protein‐complex subunits tend to be co‐expressed [42]‐[44], co‐regulation on the protein level has not been shown for large‐scale data sets. This is important since a high degree of variation in subunit stoichiometries has been demonstrated for nuclear complexes, for example, see [45]. Also, little is known about the individual contributions of transcriptional and post‐transcriptional mechanisms ensuring stoichiometric protein expression ratios.

**Figure 6:**
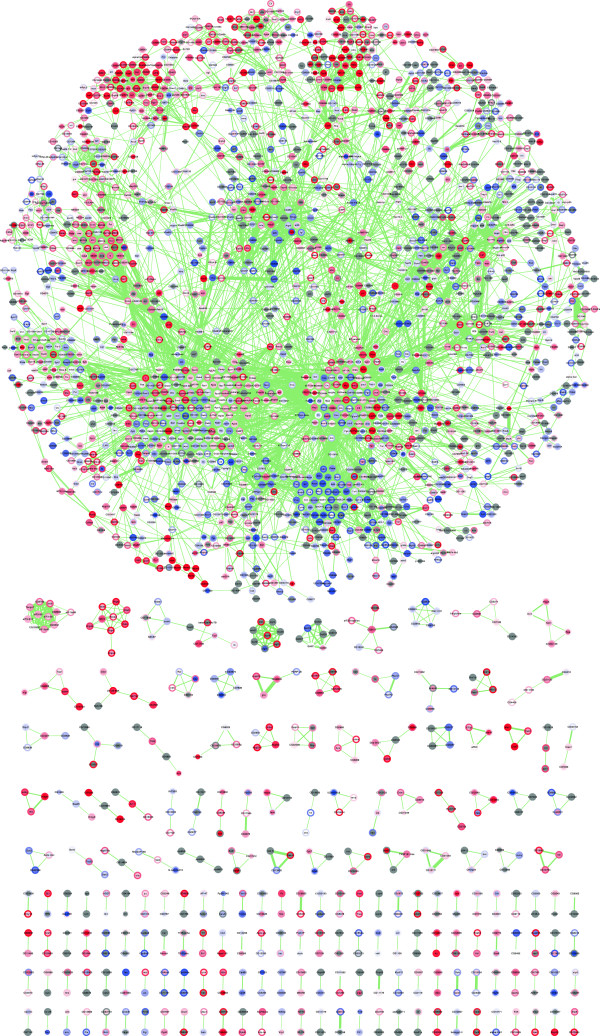
**Global protein interaction map integrated with mRNA and protein expression changes.** Visualization of mRNA and protein expression changes for all *Drosophila* protein interaction map clusters [39] using Cytoscape. Clustered proteins tend to be co‐regulated. The centers of the nodes indicate protein expression changes and the borders of the nodes mRNA expression changes. Blue represents downregulation, red represents upregulation and the color intensity is proportional to the level of regulation. Transcripts and proteins not quantified are shown in gray. Protein interactions are depicted as light green lines and their thickness is proportional to the interaction strength. See Additional file 4 for details.

We therefore used our quantitative transcriptome and proteome data to investigate the co‐regulation of protein‐complex subunits. As a reference, we used all protein interactions and complexes identified by affinity purification of tagged proteins coupled with mass spectrometry and defined by the *Drosophila* protein interaction map (DPiM) [39]. Expression changes of transcripts and proteins were mapped onto the interaction network and visualized with Cytoscape [46]. Using this integrated protein interaction network (Figure 6 and Additional file 4), we observed a clustering of co‐regulated genes into distinct areas corresponding to different protein complexes.

**Figure 7:**
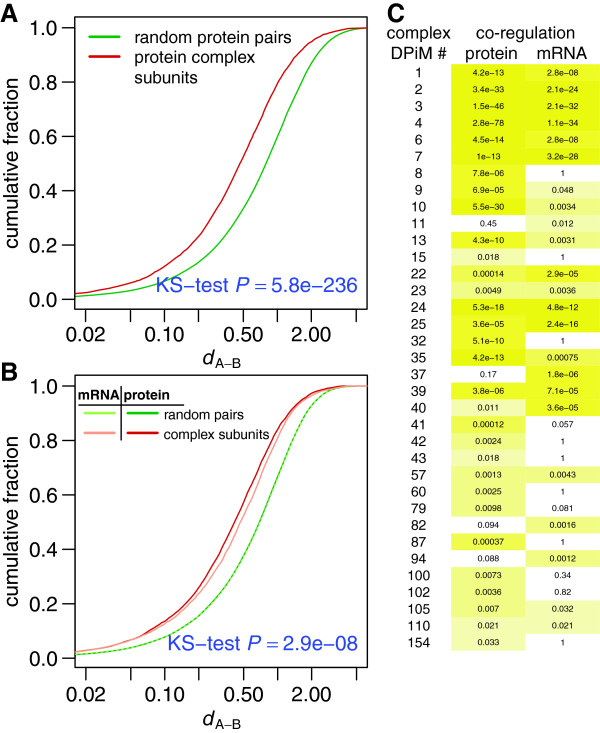
**Post‐transcriptional improvement of complex subunit co‐regulation. (A)** Subunits of annotated protein complexes (red) are significantly more co‐regulated than random protein pairs (green). **(B)** Subunits of protein complexes are significantly more co‐regulated on the protein level (red) than on the transcript level (light red). The random protein pairs are indicated in dark green, random transcript pairs in light green. **(C)** Matrix of *P* values of significantly co‐regulated protein complexes (DPiM) [39] at the mRNA level and at the protein level. The complexes are numbered according to DPiM since several of them have neither names nor known biological functions. The individual complex members are listed in Additional file 4. The color intensity indicates increasing significance. *P* values were determined by the Kolmogorov–Smirnov test (KS‐test) and corrected for multiple testing. DPiM, *Drosophila* protein interaction map.

To confirm this co‐regulation quantitatively, we determined the similarity of regulation between pairs of genes *A* and *B* by calculating the absolute difference of their *z*‐transformed log_2_‐fold expression changes *d*_*A*−*B*_. Overall, we found that interacting proteins within a complex exhibited significantly higher co‐regulation (that is, smaller *d*_*A*−*B*_) than randomized protein pairs (Figure 7A). We observed a qualitatively similar effect for the transcripts (Additional file 5: Figure S5), but the co‐regulation was significantly stronger at the protein than at the mRNA level (Figure 7B). Taken together, our data indicate that despite co‐regulation of complex subunits on the transcript level, significant fine‐tuning of protein stoichiometry occurs post‐transcriptionally.

### Only 11% of protein complexes exhibit significant subunit co‐regulation

In general, it is assumed that protein‐complex subunits are co‐regulated and maintain stable stoichiometric compositions. However, the distinction between permanent and transient complexes based on mRNA co‐expression in yeast [44] and the discovery of variable subunit compositions for some nuclear complexes [45] indicate that there are exceptions to this rule.

To determine if the co‐regulation we observed on the global level is due to co‐regulation of all or of only a subset of protein complexes, we set out to identify the individual complexes that were co‐regulated between wild‐type and *brat* samples. For this, we compared the co‐regulation of subunits of annotated protein complexes with randomly assembled ‘complexes’. Surprisingly, we found that only 23 of 274 complexes were co‐regulated on the transcript level. On the protein level, however, co‐regulation was stronger and we identified 31 complexes exhibiting significant co‐regulation (Figure 7C), supporting our conclusion for the post‐transcriptional adjustment of protein stoichiometries and the higher importance of protein versus mRNA expression control.

The low fraction of co‐regulated complexes (11% on the protein level) indicates that either co‐regulation is not a general feature of all protein complexes, or, more likely, that most complexes found in one biological system/state do not necessarily exist in other systems/states or exhibit different subunit compositions [45]. In addition, different molecules of a protein could be subunits of different protein complexes at a time.

As all annotated complexes were isolated from cultured cells of late embryonic origin (S2R+ cells) [39], and, since they exhibited high co‐regulation in adult brain tissue, we propose to classify them as permanent core complexes of *Drosophila*. Complexes like the proteasome (DPiM #4), the SNAP/SNARE complex (DPiM #7), the eukaryotic initiation factor 3 complex (eIF3, DPiM #24) and the ATP synthase complex (DPiM #25) fall into this category, both at the mRNA level and at the protein level, whereas the exosome (DPiM #41), the prefoldin complex (DPiM #42), the TCP‐1 ring complex or chaperonin‐containing TCP‐1 complex (TRiC/CCT, DPiM #32) and the minichromosome maintenance complex (MCM, DPiM #60) are exclusively co‐regulated on the protein level.

Given this high variability, we would like to suggest an extension of the concept of permanent and transient complexes introduced by [44] because higher organisms are characterized by having different tissue types and specific developmental programs. In this situation, many more complexes have to be characterized as dynamic or transient, since they might only occur at specific times, places or physiological states during an organism’s lifetime.

### Characterization of individual complexes in a tumor based on subunit co‐regulation

In total, our analysis defined 31 co‐regulated core complexes (Figure 7C). From those, we selected a subset of well‐known complexes and manually re‐analyzed their annotated subunit composition as well as their potential for tumorigenesis taking into account expression changes at the mRNA level and at the protein level.

**Figure 8:**
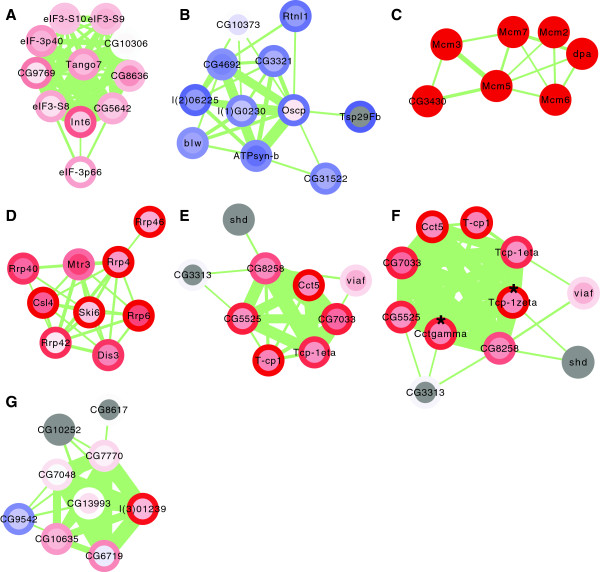
**Examples of co‐regulated protein complexes. (A)** Eukaryotic initiation factor 3. **(B)** ATP synthase complex. **(C)** MCM complex. **(D)** Exosome. **(E)** TRiC/CCT complex according to DPiM. **(F)** TRiC/CCT complex extended for co‐regulated subunits (indicated by asterisks). **(G)** Prefoldin complex. The centers of the nodes indicate protein expression changes and the borders of the nodes mRNA expression changes. Blue represents downregulation, red represents upregulation and the color intensity is proportional to the level of regulation. Transcripts and proteins not quantified are shown in gray. Protein interactions are depicted as light green lines and their thickness is proportional to the interaction strength. DPiM, *Drosophila* protein interaction map; MCM, minichromosome maintenance complex; TRiC/CCT, TCP‐1 ring complex or chaperonin‐containing TCP‐1 complex.

The eIF3 complex (DPiM #24, Figure 8A) is essential for the assembly of the translation initiation machinery, namely the recruitment of initiator Met‐tRNAi and mRNA to the 40S ribosome, and the subsequent scanning for the AUG start codon [47]. Aberrant mRNA and protein levels of eIF3 subunits have been detected in a wide variety of solid tumors and cancer cell lines, and eIF3 over‐expression can promote malignant transformation (see [48] and references therein). We found eIF3 mildly but consistently upregulated in brain tumors at the transcript as well as at the protein level.

The ATP synthase complex (DPiM #25, Figure 8B) is involved in the oxidative phosphorylation pathway and employs the electrochemical gradient at the inner mitochondrial membrane for generating ATP from ADP. The downregulation of oxidative phosphorylation is a well‐known metabolic hallmark of cancer cells, called the Warburg effect [49,50]. In the *brat* tumors, ATP synthase was downregulated both at the transcript level and protein level. Interestingly, the glycolytic enzyme L‐lactate dehydrogenase (ecdysone‐inducible gene L3, ImpL3 in *Drosophila*) was about twofold upregulated suggesting that *Drosophila* tumors might provide a suitable model for analyzing the causal relationships of the Warburg effect with cancer progression. Knockdown of this enzyme has been shown to increase mitochondrial respiration and to attenuate tumor growth [51,52].

The MCM complex (DPiM #60, Figure 8C) functions as a replicative helicase. It unwinds duplex DNA and enables fork progression during DNA replication [53]. We found all six complex members strongly upregulated both at the transcript level and at the protein level. Coaffinity purification identified an additional member of the MCM complex, the previously uncharacterized protein CG3430 [39]. Our data show the co‐regulation of CG3430 with the other six MCM complex members, hence supporting this assignment.

The exosome complex (DPiM #41, Figure 8D) is required for 3’→5’ RNA processing and turnover [54]. All its subunits were upregulated; however, the upregulation was generally stronger on the mRNA level than on the protein level. Together with the observation that the exosome subunits are significantly co‐regulated at the protein but not the transcript level (Figure 7C), this suggests, that post‐transcriptional mechanisms might be involved in regulating exosome expression. It has been shown that the exosome interacts and co‐localizes with the essential elongation factor Spt6 at active chromatin, indicating that the exosome might exert its pre‐mRNA surveillance function co‐transcriptionally [55]. Our data support this interaction by demonstrating the co‐regulation of Spt6 with the exosome.

The TRiC/CCT complex (DPiM #32, Figure 8E) is an essential, ATP‐dependent chaperonin consisting of two identical stacked rings with eight paralogous subunits per ring. It interacts with about 10% of newly synthesized cytosolic proteins and prevents the accumulation of toxic aggregates [56,57]. Guruharsha *et al.* identified six of the eight known TRiC/CCT complex members plus three weakly connected proteins (CG3313, shd, viaf), whereas the remaining two core subunits, CCT *γ* and Tcp‐1 *ζ*, were assigned to the DPiM clusters #8 and #28, respectively [39]. According to our mRNA and protein data, however, the co‐regulation of CCT *γ* and Tcp‐1 *ζ* was most consistent with them belonging to the TRiC/CCT complex (Figure 8F). For the three weakly associated proteins, we were not able to detect CG3313 nor shd, but viaf was co‐regulated with the other complex members. Consistent with the upregulation of the TRiC/CCT complex in brain tumors, we have previously shown that tissue‐specific knockdown of subunits by RNAi leads to under‐proliferation or death of neuroblasts [58].

The TRiC/CCT complex is recruited to nascent chains by the multisubunit chaperone complex prefoldin (DPiM #42, Figure 8G) allowing co‐translational folding of proteins [57]. Three new subunits have been proposed for the prefoldin complex: CG8617, CG9542 and CG10252 [39]. We were not able to identify CG8617 nor CG10252 on the protein level, and CG9542 exhibited a strong downregulation unlike the behavior of the established complex members. Therefore, we propose that these prefoldin interaction partners are cell‐type‐specific subunits of the complex and are most likely not an integral part of the prefoldin complex in adult female fly heads.

Taken together, we have shown that co‐regulation data at the mRNA level and at the protein level provide valuable additional information for protein‐complex assignment, especially if protein‐complex data is to be used for different experimental systems.

## Conclusions

We have compiled a comprehensive data set of tissue‐specific expression changes that occur in a tumor model both on the transcriptome and on the proteome level (Additional file 6). In our integrative analysis we use this data set and demonstrate the impact of post‐transcriptional gene regulation for different biological processes and protein complexes.

To achieve iTRAQ quantification for 70% of all expressed protein‐coding genes in a complex tissue, we employed: (1) digestion with two proteases to produce largely non‐overlapping peptides, (2) high‐resolution chromatography and fractionation to reduce sample complexity and (3) different mass spectrometry fragmentation techniques to obtain optimal quantitative information. This protocol is in principle applicable to clinical samples, since it does not require *in vivo* labeling. Our data set covers 86% of the expressed proteins in the *Drosophila* head annotated to distinct biological pathways (Figure 3C).

At the global level, we provide evidence for a general regulatory function of the transcript UTRs, that is, shorter 3’ and 5’ UTRs lead to increased protein upregulation. This finding indicates that regulatory elements in the UTRs ensure proper protein expression control and that transcripts with longer UTRs are less prone to misexpression, potentially due to the dampening presence of binding sites for RNA‐interacting proteins or miRNAs.

Overall, alterations in transcript and protein expression are well correlated. However, distinct biological processes show highly different correlations. This suggests that post‐transcriptional regulation strongly affects some processes like transcription whereas other processes like nucleotide metabolism are barely affected (Figure 5). The differential effects of post‐transcriptional regulation in wild‐type and tumor tissue should be taken into account when analyzing transcriptome data and, in addition, might offer new directions for targeted tumor treatment.

By integrating our data set with protein‐complex information [39], we have compiled one of the first systems‐level networks for the dynamics of protein complexes (Figure 6). By statistically investigating the co‐regulation of protein‐complex subunits we show that complex stoichiometry is ensured by both transcriptional and post‐transcriptional contributions, and that co‐regulation on the protein level is more stringently controlled than on the mRNA level. The function of many complexes critically depends on the proper stoichiometric presence of all subunits, and the consequences of the misexpression of any one subunit can range from wasting energy to dominant negative effects and diseases. Therefore, this two‐tiered mechanism is important for controlling the relative abundance of protein‐complex subunits. Surprisingly, however, we find that only a small fraction of protein‐complex subunits is co‐regulated in the *Drosophila* brain suggesting that complexes exhibit high degrees of context‐dependent dynamics.

The analysis of our integrative network shows that several protein complexes are consistently deregulated in tumors, and we find preliminary evidence for alterations reminiscent of the Warburg effect (Figure 8B). We provide examples for which the co‐regulation of potential protein‐complex subunits contributes valuable additional information for assigning subunits to the correct complexes. Since large‐scale protein interaction analyses are usually performed in cell culture systems, our approach could be used to re‐evaluate this information in a tissue‐specific context.

In summary, besides providing a valuable resource for further system‐wide studies and quantitative biology, our data offers novel insights into characteristic alterations of tumor gene expression and post‐transcriptional gene regulation.

## Materials and methods

### Sample preparation

All *Drosophila* stocks were maintained and crossed at 22°C according to standard procedures. The *brat* allele *brat*^*k*06028^ was acquired from the Bloomington *Drosophila* Stock Center (Indiana) and balanced over *CyO*, *P{hs‐hid}* to allow us to select homozygous mutant offspring using a heat shock. For transcriptome and proteome analysis, adult female flies that were 1 to 3 days old were collected manually (wild‐type control and *brat*^*k*06028^). Flies were transferred into 15 ml conical tubes and snap‐frozen in liquid nitrogen. Heads were separated by vigorously shaking and vortexing the tubes for 30 s and then applying the mixture to a stack of sieves (800, 590, 355 µm mesh openings) submerged in liquid nitrogen. The isolated heads were transferred into microfuge tubes and stored at −80°C for further analysis. Fly heads were ground to a powder using a mortar and pestle cooled with liquid nitrogen.

### Transcriptome sequencing

Total RNA was isolated from adult fly heads by TRIzol purification (Invitrogen), and genomic DNA was removed using gDNA eliminator columns from the RNeasy Mini Kit (Qiagen) following the manufacturer’s instructions. RNA quality was assessed by spectrophotometry (NanoDrop, Thermo Fisher Scientific) and on a Bioanalyzer (Agilent). The RNA was enriched for poly(A)+ mRNA (Dynabeads mRNA purification kit, Invitrogen), fragmented and subjected to first‐strand cDNA synthesis (based on a protocol by [59]). After second‐strand cDNA synthesis, double‐stranded cDNA was purified and quantified. The library was prepared using a modified protocol from Illumina with NEBNext DNA sample Prep Reagent kits (NEB). Double‐stranded cDNA was end‐repaired, poly(A) was added and adapters were ligated to DNA fragments. After size selection (200 to 600 bp), and UDGase‐treatment for strand specificity, adapter‐modified DNA fragments were enriched by PCR. Next, 76‐base paired‐end sequencing was performed on a Genome Analyzer IIx (Illumina).

The strand‐specific paired‐end reads were screened for ribosomal RNA by alignment (maximum of three mismatches) against known rRNA sequences (RefSeq) using Bowtie [60]. The insert statistics were estimated by aligning the remaining reads uniquely to the transcriptome and calculating the mean insert length and standard deviation. The rRNA‐subtracted paired‐end reads were aligned with TopHat [61] against the *Drosophila melanogaster* genome (release 5). Introns of 30 to 150,000 bp were allowed based on FlyBase statistics. Maximum multihits was set to 1, and microexon‐search was enabled. Additionally, a gene model was provided as gene transfer format (GTF) file (Ensembl BDGP5.25.60). Aligned reads in valid pairs were subjected to FPKM estimation using Cufflinks [62,63]. Bias detection and correction were performed in this step. Furthermore, only those fragments compatible with Ensembl annotation (BDGP5.25.60) were allowed and counted towards the number of mapped hits used in the FPKM denominator.

The transcriptome data have been deposited in the NCBI Gene Expression Omnibus and are accessible through GEO Series accession number GSE51412.

### Protein digestion and peptide iTRAQ labeling

Fly‐head powder was resuspended in 8 M urea/0.1 M triethylammonium bicarbonate (TEAB) and centrifuged to remove insoluble material. Proteins in the supernatant fraction were precipitated by the addition of ice‐cold acetone and incubated at −80°C overnight. After centrifugation, pellets were resuspended in iTRAQ dissolution buffer (0.5 M TEAB) containing 1% RapiGest (Waters), and the protein concentration was determined using the BCA Protein Assay Kit (Pierce). Cysteine residues were reduced and alkylated using tris‐(2‐carboxyethyl)phosphine (TCEP) and methyl methanethiosulfonate (MMTS) according to the instructions for the iTRAQ labeling kit (Applied Biosystems). Samples were digested with trypsin or LysC at 37°C for 16 h and labeled separately with iTRAQ 4‐plex reagents according to the manufacturer’s instruction, using 100 µg peptides for each label. A duplicate labeling strategy was pursued, that is, each sample was labeled with two different tags.

### Two‐dimensional fractionation of labeled peptides

Labeled peptides from four samples were combined, acidified with formic acid (FA), lyophilized, resuspended in 5 mM sodium phosphate buffer (pH 2.7) containing 15% acetonitrile (ACN), and injected onto a Polysulfoethyl‐A 3 µm (PolyLC), 25 cm × 1 mm inner diameter (i.d.) column. Separation was performed on an UltiMate nano LC system (Dionex, Thermo Fisher Scientific) at a flow rate of 50 µl/min using the following gradient: 20 min 100% A, followed by a linear gradient to 10% B/50% C in 80 min, 25% B/50% C in 10 min, 50% B/50% C in 5 min and maintained for a further 15 min, then within 5 min to 100% A for column re‐equilibration (A: 5 mM sodium phosphate buffer, 15% ACN, pH 2.7; B: 5 mM sodium phosphate buffer, 0.5 M NaCl, 15% ACN, pH 2.7; C: 5 mM sodium phosphate buffer, 15% ACN, pH 6.0). One‐minute fractions were collected and fractions with a low peptide content were pooled. Samples were concentrated in a vacuum centrifuge concentrator to remove ACN and diluted in 0.1% trifluoroacetic acid (TFA). Reversed phase separation was performed on an UltiMate 3000 RSLCnano high performance liquid chromatography (HPLC) system (Dionex, Thermo Fisher Scientific). After injection, samples were concentrated and desalted on a trapping column (AcclaimPepMap 3 µm, 100 Å, 2 cm × 75 µm i.d.) using 0.1% TFA at a flow rate of 5 µl/min as a loading solution, and then separated on an analytical column (AcclaimPepMap 2 µm, 100 Å, 25 cm × 75 µm i.d.) using a linear gradient from 2% to 25% B in 175 min, then to 90% B in 5 min, maintained for 5 min, then within 2 min to 100% A for column re‐equilibration (A: 2% ACN/0.1% FA; B: 80% ACN/10% trifluoroethanol/0.08% FA) at a flow rate of 275 nl/min. The HPLC was directly coupled online to a LTQ‐Orbitrap Velos instrument (Thermo Fisher Scientific) via a nanoelectrospray source (Proxeon, Thermo Fisher Scientific).

### Shotgun mass spectrometry

The LTQ‐Orbitrap Velos instrument was operated in positive ionization mode. The source voltage was set to 2.0 kV, transfer tube temperature was 250°C and the S‐lens radio frequency (RF) level was set to 68%. A mass spectrometry (MS) survey scan was performed in the Orbitrap from a mass‐to‐charge ratio (*m*/*z*) of 350 to 2,000 at a resolution of 60,000. The automatic gain control (AGC) target value was set to 1,000,000 ions and the maximum fill time was 500 ms. The lock mass option was enabled using the dimethylcyclosiloxane background ions (protonated [(CH_3_)_2_SiO]_6_; *m*/*z*=445.120025) for internal calibration. The MS survey scan was followed by 12 data‐dependent scans.

The six most abundant ions excluding singly charged ions were selected for fragmentation. For each selected precursor ion, two tandem mass spectra were obtained: one spectrum was acquired in the ion trap for maximum sensitivity of identification and the other spectrum was acquired in the Orbitrap at a resolution of 7,500 (AGC target 100,000, maximum fill time 250 ms) for precise quantification. The strategy is analogous to the previously described method, which combined a CID spectrum acquired from the ion trap with a HCD spectrum acquired from the Orbitrap [28]. However, for samples digested with trypsin a CID spectrum was recorded in the ion trap, while for samples digested with LysC an ETD spectrum was recorded using the ion trap. For CID spectra, the AGC target was set to 6,000 ions, maximum fill time was 200 ms, activation time was 10 ms, normalized collision energy was 35%, and multistage activation was activated using the following neutral loss *m*/*z* list: 32.6, 49.0 and 98.0. For ETD spectra, the AGC target was set to 10,000 ions, maximum fill time was 200 ms, supplemental activation was enabled, and the reaction time was set to 120 ms for doubly charged precursor ions and 80 ms for triply charged precursor ions, reduced in a charge‐dependent manner for higher charged precursor ions. Reagent ion target was 300,000 ions with a maximum fill time of 80 ms for ETD, and the reagent ion source chemical ionization (CI) gas pressure was tuned whenever indicated to ensure adequate fluoranthene signal. For HCD spectra, a stepped collision energy was employed with two steps at 42% and 58% normalized collision energy to permit both identification and quantification.

To fragment peptides close to the apex of the elution signal, the chromatography feature was activated using a correlation of 0.8 and an expected peak width of 10 s. In all cases, one microscan was recorded. The isolation window was 2.4 *m*/*z* for spectra recorded in the ion trap and 1.6 *m*/*z* for spectra recorded in the Orbitrap to minimize interference with iTRAQ quantification by precursor ions with similar *m*/*z*. To avoid oversampling, the *m*/*z* values of precursor ions selected for fragmentation were subsequently excluded for 180 s using a dynamic exclusion window of ±5 ppm, with the early expiration feature deactivated. Database searches were performed on both ion trap and Orbitrap tandem mass spectra for identification while quantification was based on iTRAQ reporter ions extracted from Orbitrap tandem mass spectra. Measurements were started with SCX fractions separated from one another by 10 min of elution time during strong cation exchange separation. Subsequently, adjacent SCX fractions were measured using exclusion lists of peptides identified in adjacent SCX fractions to maximize proteome coverage. The exclusion lists were based on a retention time window of 2 min before and 4 min after the retention time of the peptide identified in the adjacent SCX fraction, the exact theoretical *m*/*z* of the identified peptide and a tolerance window ±7.5 ppm. To make efficient use of the exclusion lists, monoisotopic precursor selection was enabled and preview mode was deactivated.

The mass spectrometry proteomics data have been deposited with the ProteomeXchange Consortium [64] via the PRIDE partner repository [65] with the data set identifier PXD000478.

### Data analysis

Data generated by LC‐MS/MS analysis were searched against a database containing a translation of all open reading frames in FlyBase (r5.25) [66] and common contaminants, concatenated to a reversed decoy database so that the FDR could be estimated using the target‐decoy strategy [67]. Proteome Discoverer (version 1.3.0.211, Thermo Fisher Scientific) was used as a search engine interface for Mascot [68], Sequest [69], X!‐Tandem [70] and ZCore [71]. Oxidation of methionine was set as dynamic modification, and methylthio (C) and iTRAQ4plex label (K, N‐terminus) as static modifications. The minimal peptide length was set to seven amino acids, and a maximum of two missed cleavages was allowed for trypsin‐ and LysC‐digested samples. To allow for an integrative analysis of transcriptome and proteome data, protein level changes were determined using only peptides that mapped unambiguously to one gene. Peptides that could be derived from proteins encoded by different gene models (‘shared peptides’) were excluded [72]. Furthermore, only peptides that showed less than a twofold difference between duplicate iTRAQ channels were included in the analysis. Peptide identifications from different search engines were combined using a modified version of the combined FDR score [73]. Reporter ion intensities were corrected for isotope impurities in the iTRAQ labels. To account for the error structure and stabilize the variance of the reporter ion intensities, a variance stabilizing transformation was applied [74]. Protein ratios were calculated as the 20% trimmed mean from the median‐centered peptide ratios [74]. Proteins were filtered for a maximum FDR of 5% [75].

### Selected reaction monitoring assays

To validate iTRAQ quantification using an independent label‐free method, SRM assays were performed on selected proteins. Suitable peptides were selected from either the iTRAQ data set or the Peptide Atlas [76] or were predicted *in silico*. The selection process was aided by MRM Pilot Software (AB Sciex). In addition, three peptides each from four different proteins (fructose‐bisphosphate aldolase [UniProt:P07764], heat shock protein 70 kDa [UniProt:P11147], enolase [UniProt:P15007] and phosphoglycerate kinase [UniProt:Q3KN29]) that were found unregulated in the iTRAQ data set were used to normalize the runs from wild‐type and *brat* samples. Unlabeled protein extracts were separated by one‐dimensional reversed‐phase nanoHLPC on an Ultimate 3000 (Thermo Fisher Scientific). Samples were loaded onto a trapping column (PepMap C18, 5 µm, 100 Å, 5 mm × 0.3 mm i.d.) using 0.1% TFA at a flow rate of 20 µl/min and desalted for 20 min. Peptides were separated on a 250 mm × 75 µm i.d. analytical column (PepMap C18, 3 µm, 100 Å) at a flow rate of 300 nl/min by applying the following gradient: in 130 min from 0 to 100% B, in 30 min to 100% C, held for 5 min at 100% C before re‐equilibration with 100% A (A: 5% ACN, 0.1% FA; B: 30% ACN, 0.1% FA; C: 80% ACN, 10% 2,2, 2‐trifluoroethanol, 0.08% FA). The nanoLC was directly coupled to a QTRAP4000 hybrid triple quadrupole/linear ion‐trap instrument (Applied Biosystems). Transitions were validated via MS2 spectra and the best two to three transitions per peptide were selected for quantification. Peptides that could not be verified by MS2 were synthesized in‐house on a Syro Peptide Synthesizer (MultiSyntech) and used as standards to determine retention time and optimal transitions. For quantification runs, 2 µg of unlabeled protein extract was injected and the mass spectrometer was operated in SRM mode without acquisition of MS2 spectra. Transitions were monitored either with a fixed dwell time of 100 ms for candidate proteins and 50 ms for normalization peptides, or by scheduled SRM. Peak area integration was done with the MultiQuan 1.0 software (Applied Biosystems).

### Bioinformatics

All statistical analyses were performed using the R programming language [77]. Primary sequence features like codon adaptation index, molecular weight and isoelectric point were calculated using EMBOSS applications [78]. Hydrophobicity was calculated according to [79]. For every gene, the longest FlyBase‐annotated 5’ UTR, coding sequence and 3’ UTR were used. The hypergeometric test was applied for KEGG enrichment analysis and corrected for multiple testing.

To determine if the correlations between transcript and protein level changes for different biological pathways were significantly different from the global correlation, random sampling experiments were performed with the sample size matching the number of proteins quantified in each pathway. For every pathway, a skew‐normal distribution was fitted to the Spearman correlation coefficient distribution of 10,000 random samples and the *P* value was estimated [80].

To control for the different spread of regulation in different pathways on the transcript or protein level, the gene list was rank‐ordered according to transcript or protein level change, respectively, and random samples were drawn out of the 20 closest neighbors of each gene in the pathway under investigation, thereby maintaining the pathway‐specific range of regulation. The correlation coefficients of 10,000 random samples were calculated and a skew‐normal distribution was fitted to estimate the *P* value.

Transcript and protein level changes were expressed as log_2_‐fold changes and *z*‐transformed. For co‐regulation analysis, transcript and protein *z* values were quantile normalized. Co‐regulation between two genes *A* and *B* was expressed as the absolute difference between their normalized *z* values *d*_*A*−*B*_, with small values indicating co‐regulation. This value measured the co‐regulation of complex subunits irrespective of their stoichiometric composition. We define a protein complex as consisting of at least three different subunits, and we distinguish two categories of protein–protein interactions: random protein pairs that generally do not interact and interacting proteins that are members of the same protein complex.

## List of abbreviations

ACN, acetonitrile; AGC, automatic gain control; bp, base pair; CDS, coding sequence; CID, collisional‐induced dissociation; DPiM, *Drosophila* protein interaction map; eIF3, eukaryotic initiation factor 3; ETD, electron transfer dissociation; FA, formic acid; FDR, false discovery rate; FPKM, fragments per kilobase of transcript per million mapped fragments; HCD, higher energy C‐trap dissociation; iTRAQ, isobaric tag for relative and absolute quantification; KEGG, Kyoto Encyclopedia of Genes and Genomes; LC‐MS/MS, liquid chromatography‐tandem mass spectrometry; MCM, minichromosome maintenance complex; miRNA, microRNA; PCR, polymerase chain reaction; rRNA, ribosomal RNA; SCX, strong cation exchange; SRM, selected reaction monitoring; TEAB, triethylammonium bicarbonate; TRiC/CCT, TCP‐1 ring complex or chaperonin‐containing TCP‐1 complex; UTR, untranslated region.

## Competing interests

The authors declare that they have no competing interests.

## Authors’ contributions

CJ and JAK conceived the project. CJ performed most of the experiments and analyzed the data. ID and PP generated the proteomics data. HH generated the transcriptome data. GA and KM designed and supervised the mass spectrometry experiments. RS developed the chromatography procedure. CJ and JAK wrote the manuscript. All authors read and approved the final manuscript.

## Supplementary Material

Additional file 1 **Figure S1.** iTRAQ reproducibility. (A) Technical reproducibility of iTRAQ protein quantification. (B) Technical reproducibility between iTRAQ and label‐free SRM protein quantification. Error bars indicate standard deviations. (C) Reproducibility of protein level changes between biological replicates measured with SRM and iTRAQ. Error bars indicate standard deviations. (D) Venn diagram showing the number of quantified unique peptides in the trypsin‐ and LysC‐digested samples. The samples were largely complementary: only 16% of the quantified peptides were identical. (E) Venn diagram showing the number of quantified proteins from the trypsin and LysC samples. This shows that 75% of the proteins were quantified in both samples. (F) Correlation of iTRAQ protein quantification using either trypsin‐ or LysC‐digested samples. Click here for file

Additional file 2 **Figure S2.** Analysis of proteome coverage. For each bin the number of annotated (dark gray), expressed (light gray) and quantified proteins (white) are shown together with the percentage of quantified proteins (red). (A) Proteome coverage is higher for proteins predicted by the codon adaptation index to be more abundant. The blue line indicates the percentage of quantified proteins from all annotated protein. (B) Proteome coverage is higher for larger proteins. (C) Proteome coverage is lower for very hydrophobic proteins. (D) Proteome coverage is higher than 60% for all isoelectric points. Click here for file

Additional file 3 **Figure S3.** Correlation of protein level change with transcript abundance. Correlation of protein level change with transcript abundance in (A) wild‐type and (B) *brat* samples. Click here for file

Additional file 4 **Cytoscape protein interaction network.** Cytoscape file containing log_2_‐fold expression changes on mRNA and protein levels combined with DPiM protein interaction data. The centers of the nodes indicate protein expression changes and the borders of the nodes mRNA expression changes. Blue represents downregulation, red represents upregulation and the color intensity is proportional to the level of regulation. Transcripts and proteins not quantified are shown in gray. Protein interactions are depicted as light green lines and their thickness is proportional to the interaction strength. Click here for file

Additional file 5 **Figure S5.** Complex co‐regulation. Protein‐complex co‐regulation on the mRNA level. Transcripts encoding subunits of annotated protein complexes (red) are significantly more co‐regulated than random pairs (green). Click here for file

Additional file 6 **Data set of transcriptome and proteome changes.** Complete data set of transcript and protein quantification data, containing FlyBase gene number, gene name, protein level change (log_2_‐fold change), standard deviation of log_2_ protein level change, number of quantified spectra, transcript level change (log_2_‐fold change), *brat* FPKM, control FPKM, standard deviation of *brat* FPKM and standard deviation of control FPKM. Since a double‐labeling approach was performed, each quantified spectrum contains two reporter ions from both *brat* and control samples. Click here for file
