# Supplementary figures and images for: Transcriptome and proteome quantification of a tumor model provides novel insights into post‐transcriptional gene regulation
Source: Genome Biol. 2013 Nov 30;14(11):r133. doi: 10.1186/gb-2013-14-11-r133 (PMC4053992; doi:10.1186/gb-2013-14-11-r133)

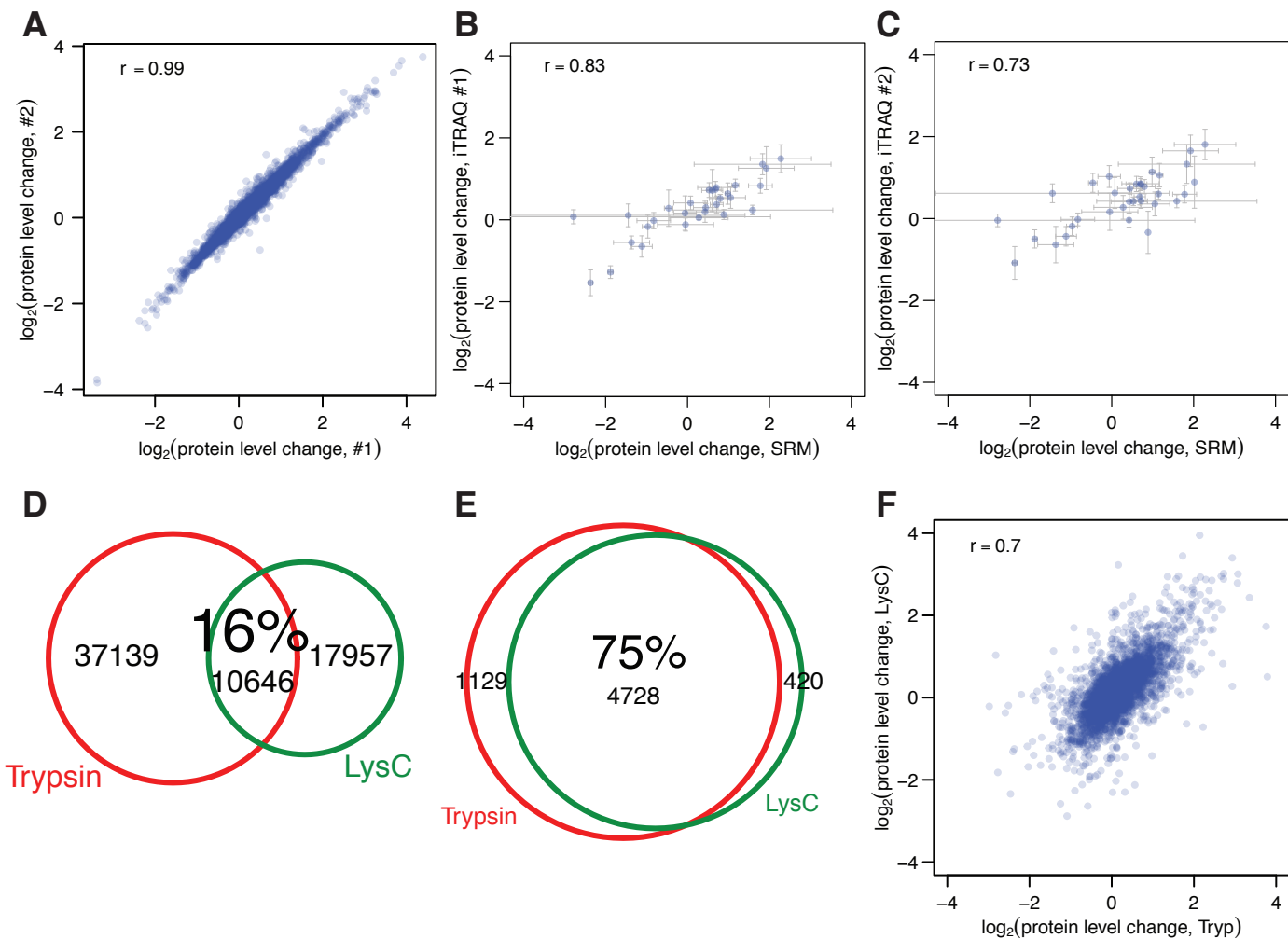

Supplement: Additional file 1 — Figure S1. iTRAQ reproducibility. (A) Technical reproducibility of iTRAQ protein quantification. (B) Technical reproducibility between iTRAQ and label‐free SRM protein quantification. Error bars indicate standard deviations. (C) Reproducibility of protein level changes between biological replicates measured with SRM and iTRAQ. Error bars indicate standard deviations. (D) Venn diagram showing the number of quantified unique peptides in the trypsin‐ and LysC‐digested samples. The samples were largely complementary: only 16% of the quantified peptides were identical. (E) Venn diagram showing the number of quantified proteins from the trypsin and LysC samples. This shows that 75% of the proteins were quantified in both samples. (F) Correlation of iTRAQ protein quantification using either trypsin‐ or LysC‐digested samples. [file gb-2013-14-11-r133-S1.PDF]

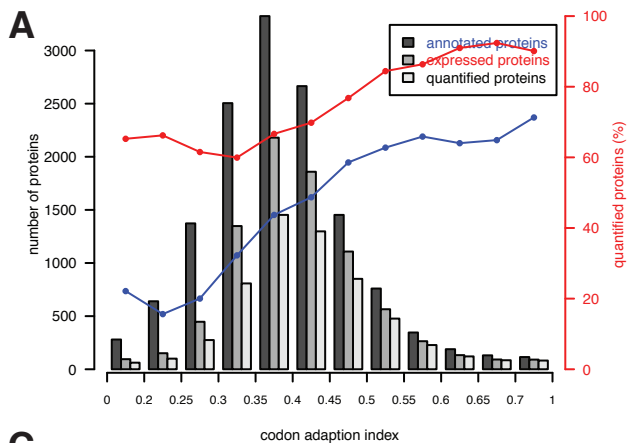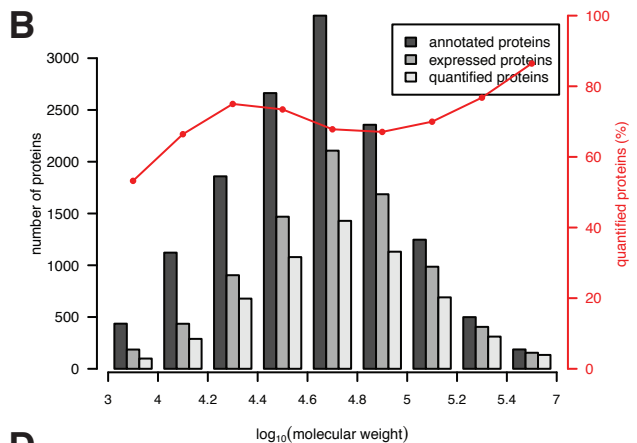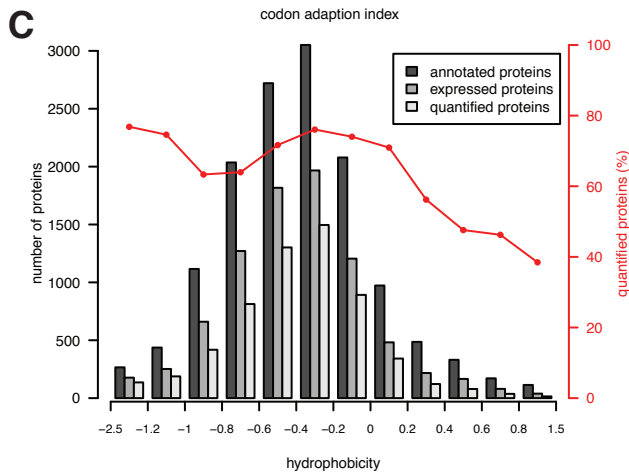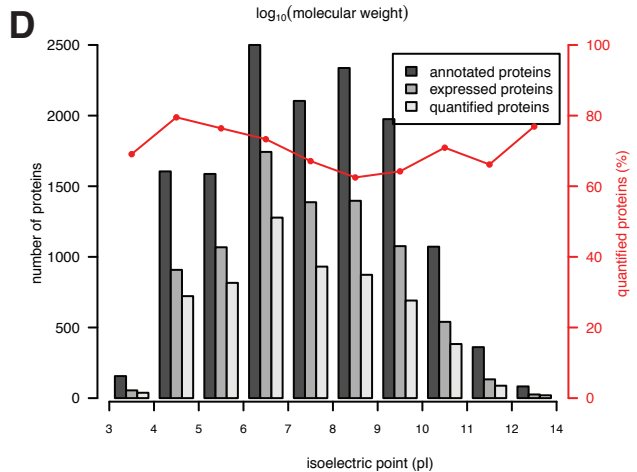

Supplement: Additional file 2 — Figure S2. Analysis of proteome coverage. For each bin the number of annotated (dark gray), expressed (light gray) and quantified proteins (white) are shown together with the percentage of quantified proteins (red). (A) Proteome coverage is higher for proteins predicted by the codon adaptation index to be more abundant. The blue line indicates the percentage of quantified proteins from all annotated protein. (B) Proteome coverage is higher for larger proteins. (C) Proteome coverage is lower for very hydrophobic proteins. (D) Proteome coverage is higher than 60% for all isoelectric points. [file gb-2013-14-11-r133-S2.PDF]

**A**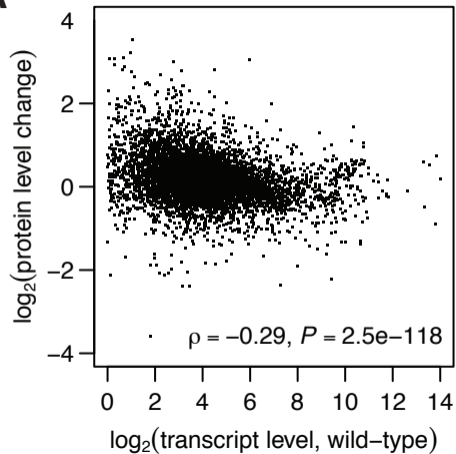**B**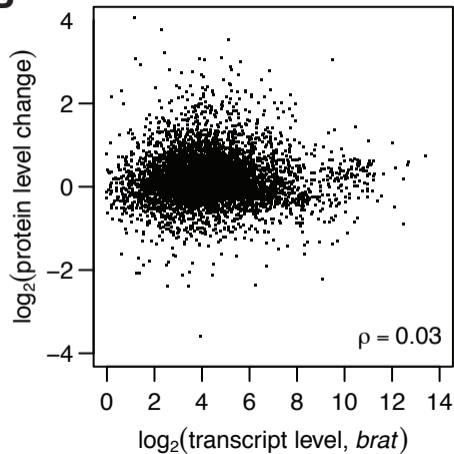

Supplement: Additional file 3 — Figure S3. Correlation of protein level change with transcript abundance. Correlation of protein level change with transcript abundance in (A) wild‐type and (B) brat samples. [file gb-2013-14-11-r133-S3.PDF]

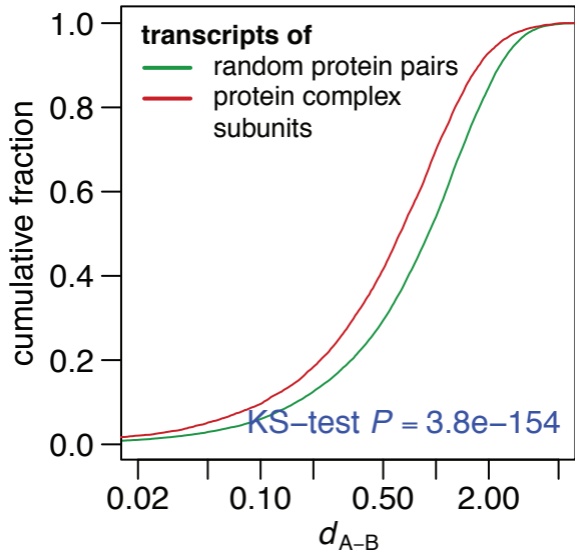

Supplement: Additional file 5 — Figure S5. Complex co‐regulation. Protein‐complex co‐regulation on the mRNA level. Transcripts encoding subunits of annotated protein complexes (red) are significantly more co‐regulated than random pairs (green). [file gb-2013-14-11-r133-S5.PDF]
